# Preclinical characterization of DEKAVIL (F8-IL10), a novel clinical-stage immunocytokine which inhibits the progression of collagen-induced arthritis

**DOI:** 10.1186/ar2814

**Published:** 2009-09-25

**Authors:** Kathrin Schwager, Manuela Kaspar, Frank Bootz, Roberto Marcolongo, Erberto Paresce, Dario Neri, Eveline Trachsel

**Affiliations:** 1 Philochem AG, c/o ETH Zurich, Institute of Pharmaceutical Sciences, Wolfgang-Pauli-Strasse 10 HCI E520, CH-8093 Zurich, Switzerland; 2 Institute of Pharmaceutical Sciences, ETH Zürich, Wolfgang-Pauli-Strasse 10, CH-8093 Zurich, Switzerland; 3 Centro Interdipartimentale Studio Biochimico-Clinico Patologie Osteoarticolari, Via Doninzetti 7, University of Siena, 53100 Siena, Italy; 4 Department of Rheumatology, Instituto Ortopedico Gaetano Pini, via Pini 9, 20122 Milan, Italy

## Abstract

**Introduction:**

In this article, we present a comparative immunohistochemical evaluation of four clinical-stage antibodies (L19, F16, G11 and F8) directed against splice isoforms of fibronectin and of tenascin-C for their ability to stain synovial tissue alterations in rheumatoid arthritis patients. Furthermore we have evaluated the therapeutic potential of the most promising antibody, F8, fused to the anti-inflammatory cytokine interleukin (IL) 10.

**Methods:**

F8-IL10 was produced and purified to homogeneity in CHO cells and shown to comprise biological active antibody and cytokine moieties by binding assays on recombinant antigen and by MC/9 cell proliferation assays. We have also characterized the ability of F8-IL10 to inhibit arthritis progression in the collagen-induced arthritis mouse model.

**Results:**

The human antibody F8, specific to the extra-domain A of fibronectin, exhibited the strongest and most homogenous staining pattern in synovial biopsies and was thus selected for the development of a fully human fusion protein with IL10 (F8-IL10, also named DEKAVIL). Following radioiodination, F8-IL10 was able to selectively target arthritic lesions and tumor neo-vascular structures in mice, as evidenced by autoradiographic analysis and quantitative biodistribution studies. The subcutaneous administration route led to equivalent targeting results when compared with intravenous administration and was thus selected for the clinical development of the product. F8-IL10 potently inhibited progression of established arthritis in the collagen-induced mouse model when tested alone and in combination with methotrexate. In preparation for clinical trials in patients with rheumatoid arthritis, F8-IL10 was studied in rodents and in cynomolgus monkeys, revealing an excellent safety profile at doses tenfold higher than the planned starting dose for clinical phase I trials.

**Conclusions:**

Following the encouraging preclinical results presented in this paper, clinical trials with F8-IL10 will now elucidate the therapeutic potential of this product and whether the targeted delivery of IL10 potentiates the anti-arthritic action of the cytokine in rheumatoid arthritis patients.

## Introduction

The therapeutic potential of recombinant cytokines is often limited by severe toxicities, even at low doses, thus preventing dose escalation and the establishment of a sufficient concentration at target tissues. It is becoming increasingly clear that monoclonal antibodies could be used to deliver cytokines at sites of disease, therefore increasing their potency and sparing normal tissues. This pharmacodelivery strategy has been mainly investigated for cancer therapy applications, leading to the preclinical [1-5] and clinical [6,7] investigation of several antibody-cytokine fusion proteins. For example, our group has brought immunocytokines based on human IL2 [8-11] and on human TNF [11-13] to phase I and phase II clinical trials.

Recently, we have observed that antibody-based pharmacodelivery strategies can also be used in the non-oncological setting [14,15]; for example, aiming at the targeted delivery of anti-inflammatory cytokines at sites of inflammation. We have reported that the L19 antibody, specific to the alternatively spliced extra-domain B (EDB) of fibronectin [16,17], could be fused to human IL10, thus generating an immunocytokine capable of preferential accumulation at neovascular sites of cancer and arthritis and capable of inhibiting the progression of established collagen-induced arthritis (CIA) in the mouse [18]. Our preclinical and clinical experience has shown that recombinant antibody fragments (e.g., single chain variable fragments (scFv) with long [19] or short [20] linkers) were particularly suited for the development of antibody-based therapeutics capable of selective accumulation at sites of disease, while being rapidly cleared from other body locations [3,21-26]. Furthermore, components of the modified extracellular matrix, such as splice isoforms of fibronectin and tenascin-C (TnC), were found to be ideal for antibody-based pharmacodelivery applications, in view of their abundant expression at accessible sites of tissue remodeling, while being undetectable in most normal human tissues [27,28].

IL10 is a particularly attractive anti-inflammatory cytokine for arthritis treatment, which has exhibited an excellent tolerability profile in rodents, monkeys and patients at doses up to 25 μg/kg [29,30]. Recombinant human IL10 (Tenovil TM) was shown to inhibit paw swelling and disease progression in the mouse CIA model. This product was also found to synergize with TNF-blocking antibodies [31] and has been tested in clinical trials in combination with methotrexate [32,33]. The clinical development of Tenovil TM was discontinued because of insufficient efficacy of the compound in humans. However, in a placebo-controlled phase I/II study American College of Rheumatology (ACR) 20 responses were 63% for the recombinant human IL10 (rhuIL10) groups, compared with 10% for placebo [32,33]. Similar results were observed with TNF blockers [34].

Encouraged by the promising results obtained with L19-IL10, we have now performed a comparative immunohistochemical analysis on synovial tissue biopsies obtained from rheumatoid arthritis patients of four extensively validated human monoclonal antibodies generated in our laboratory. In addition to L19, we studied F16 (specific to the extra-domain A1 of TnC; [10,35]), G11 (specific to the extra-domain C of TnC; [36,37]) and F8 (specific to the extra-domain A (EDA) of fibronectin; [38]). The observation of an intense and diffuse staining pattern with the anti-EDA antibody F8 led to the development of F8-IL10, a fully-human recombinant immunocytokine which is now entering clinical trials in patients with rheumatoid arthritis. In this article, we present an extensive *in vitro *and *in vivo *characterization of F8-IL10, including the ability of this therapeutic protein to preferentially localize at sites of arthritis and to inhibit disease progression in the CIA model. The clinical development plans for F8-IL10 are also justified by the excellent tolerability profile observed in rodents and monkeys.

## Materials and methods

### Immunohistochemical analysis

For immunohistochemistry on synovial tissue samples, 10 μm cryostat sections were fixed in ice-cold acetone and stained for FN-EDA, FN-EDB, TnC-A1 and TnC-C. These antibodies do not work on freshly frozen paraffin-embedded specimens. Primary antibodies in small immunoprotein (SIP) format were added onto the sections in a final concentration of 2 μg/ml and detected with rabbit anti-human IgE antibody (Dako, Glostrup, Denmark) followed by biotinylated goat anti-rabbit IgG antibody (Biospa, Milan, Italy) and streptavidin-alkaline phosphatase (SAP) complex (Biospa, Milan, Italy). Fast Red TRSalt (Sigma-Aldrich, St Louis, MO, USA) was used as the phosphatase substrate. Sections were counterstained with hematoxylin, mounted with glycergel mounting medium (Dako, Glostrup, Denmark) and analyzed with an Axiovert S100 TV microscope (Zeiss, Feldbach, Switzerland). In total, freshly frozen pathology specimens of seven patients were analyzed by immunohistochemistry.

For immunofluorescence, a double staining for FN-EDA, FN-EDB, TnC-A1 respectively TnC-C and von Willebrand factor was performed. The following primary antibodies were used: scFv(F8), scFv(L19), scFv(F16) resp. scFv(G11) and polyclonal rabbit anti-human von Willebrand factor (Dako, Glostrup, Denmark). As secondary detection antibodies mouse anti-Myc (9E10) monoclonal antibody followed by Alexa Fluor 594 goat anti-mouse IgG (Molecular Probes, Leiden, The Netherlands) was used for scFv and Alexa Fluor 488 goat anti-rabbit (Molecular Probes, Leiden, The Netherlands) for von Willebrand factor. Slides were mounted and analyzed as described before.

### Cloning, expression and characterization of a scFv(F8)-human IL10 fusion protein

The human IL10 gene was amplified from the previously cloned fusion protein L19-IL10 using the following primer sequences: a backward antisense primer, 5' TAATGGTGATGGTGATGGTGGTTTCGTATCTTCATTGTCATGTAGGCTTC-3'; and a forward sense primer, 5'-TTTTCCTTTTGCGGCCGCTCATTAGTTTC-GTATCTTCATTGTCATGTA-3', which appended part of a 15 amino acid linker (SSSSG)_3 _at its N-terminus and a stop codon and NotI restriction site at its C-terminus.

The gene for the single-chain variable fragment (F8) was amplified with a signal peptide using the following primer pair: a backward antisense primer, 5'-CCCAAGCTTGTCGACCATGGGCTGGAGCC-3' and a forward sense primer, 5'-GAGCCGGAAGAGCTACTACCCGATGAGGAAGATTTGATTTCCACCTTG-GTCCCTTG-3'. Using this strategy, a HindIII restriction site was inserted at the N-terminus and a complementary part of the linker sequence was inserted at the C-terminus.

The single-chain Fv and IL10 fragments were then assembled using PCR and cloned into the HindIII and NotI restriction sites of the mammalian cell-expression vector pcDNA3.1(+) (Invitrogen, Basel, Switzerland).

### Cloning of a TNF receptor fusion protein

TNF receptor (R) II extracellular domain was amplified using a backward antisense primer, 5'-TTTTCCTTTTGCGGCCGCTCATTA-3'; and a forward sense primer, 5'-GGGTAGTAGCTCTTCCGGCTCATCGTCCAGCGGCGTGCCCGCCAAGGTTG-3', which appended part of a 15 amino acid linker (SSSSG)_3 _at its N-terminus and a stop codon and NotI restriction site at its C-terminus.

The gene for the single-chain variable fragment (F8) was amplified with a signal peptide using the following primer pair: a backward antisense primer, 5'-CCCAAGCTTGTCGACCATGGGCTGGAGCC-3' and a forward sense primer, 5'-GAGCCGGAAGAGCTACTACCCGATGAGGAAGATTTGATTTCCACCTTG-GTCCCTTG-3'. Using this strategy, a HindIII restriction site was inserted at the N-terminus and a complementary part of the linker sequence was inserted at the C-terminus. The resulting PCR assembly product was cloned into the HindIII and NotI restriction sites of the mammalian cell-expression vector pcDNA3.1(+) expressed in CHO-S cells.

### Expression and purification of F8-IL10

CHO-S cells were stably transfected with the previously described plasmid and selection was carried out in the presence of G418 (0.5 g/l). Clones of G418-resistant cells were screened for expression of the fusion protein by ELISA using recombinant EDA of human fibronectin as antigen and protein A horseradish peroxidase for detection (GE Healthcare, Chalfont St Giles, UK). Following generation of monoclonal cell lines, the best expressing clone was adapted to growth in PowerCHO-2 CD protein-free medium (Lonza, Basel, Switzerland) for large-scale production of F8-IL10. The fusion protein could be purified from cell culture medium by protein A affinity chromatography, because there is a staphylococcal protein A binding site present on most V_H_3 subclasses [39-41]. The size of the fusion protein was analyzed in reducing and nonreducing conditions on SDS-PAGE and in native conditions by fast protein liquid chromatography gel filtration on a Superdex S-200 size exclusion column (GE Healthcare, Chalfont St Giles, UK).

### Bioactivity assay

Biological activity of human IL10 was determined by its ability to induce the IL-4-dependent proliferation of MC/9 cells [42] using a colorimetric thiazole blue (MTT) dye-reduction assay (Sigma-Aldrich, St Louis, MO, USA). In a 96-well microtitre plate, 10,000 MC/9 (murine mast cell line) (ATCC-LGC, Molsheim Cedex, France) cells/well in 200 μl of medium containing 5 pg (0.05 units)/ml of murine IL4 (eBiosciences, San Diego, CA, USA) were treated for 48 hours with varying amounts of human IL10. The human IL10 standard and fusion proteins were used at a maximum concentration of 100 ng/ml IL10 equivalents and serially diluted. To this, 10 μl of 5 mg/ml MTT was added and the cells were incubated for three to five hours. The cells were than centrifuged lysed with dimethylsulfoxide (DMSO) and read for absorbance at 570 nm.

### Collagen induced arthritis mouse model

Male DBA/1 mice (8 to 10 weeks old) were immunized by intradermal injection at the base of the tail with 150 μg of bovine type II collagen (Chondrex, Inc., Redmond, WA, USA) emulsified with equal volumes of Freund's complete adjuvant (Chondrex, Inc., Redmond, WA, USA). The procedure was repeated two weeks after the first immunization. Mice were inspected daily and each mouse that exhibited erythema and/or paw swelling in one or more limbs was assigned to an imaging or treatment study.

Arthritis was monitored defining a clinical score. Each limb was graded daily in a nonblinded fashion (0 = normal, 1 = swelling of one or more fingers of the same limb and 2 = swelling of the whole paw), with a maximum score of eight per animal [43].

### Near infrared imaging of arthritic paws

The selective accumulation of SIP(F8) in arthritic mice was tested by near-infrared imaging analysis, as described by Birchler and colleagues [44]. Briefly, SIP(F8) was labeled using Alexa750 (Molecular Probes, Leiden, The Netherlands), according to the manufacturer's recommendations, and injected into the tail vein of arthritic mice (n = 3). Mice were anaesthetized using ketamin, 80 mg/kg body weight, and medetomidine, 0.2 mg/kg body weight, and imaged in a near infrared mouse imager 24 hours after injection.

### Phosphorimage analysis of arthritic paws with radiolabeled F8-IL10

For a more detailed targeting analysis of SIP(F8) and F8-IL10 the proteins were radio-iodinated and injected intravenously or subcutaneously, respectively (150 μg protein, 7 μCi). Mice (n = 2) were sacrificed 24 hours after injection, paws were exposed to a phosphorimager screen (Fujifilm, Dielsdorf, Switzerland) for one hour and read in a PhosphorImager (Fujifilm BAS-5000, Dielsdorf, Switzerland). Data were analyzed using Aida Image Analyzer v.4.15 (Fujifilm, Dielsdorf, Switzerland).

### Quantitative biodistribution studies in tumor mice

To compare the *in vivo *targeting performance after subcutaneous and intravenous injection quantitative biodistribution analyses using radiolabeled antibody preparations were performed as described before. Briefly, purified F8-IL10 was radioiodinated with ^125^I and injected intravenously or subcutaneously into 129Sv mice (n = 4) grafted with a subcutaneous F9 tumor (150 μg, 8 μCi per mouse). Mice were sacrificed 24, 48, 72, or 96 hours after injection. Organs were weighed and radioactivity was counted using a Cobra γ counter (Packard, Meriden, CT, USA). Radioactivity content of representative organs was expressed as the percentage of the injected dose per gram of tissue (%ID/g ± standard error).

In a similar experiment a comparison of targeted and systemic application of IL10 was performed. The antibody specific to hen egg lysozyme (HyHel) 10-IL10 and F8-IL10 were labeled with ^125^I and intravenously injected into 129Sv mice (n = 4) grafted with a subcutaneous F9 tumor (150 μg, 8 μCi per mouse). Tumor and organ uptake was measured 24 hours after injection, as described above. Experiments were performed in agreement with Swiss regulations and under a project license granted by the Veterinäramt des Kantons Zürich, Switzerland (169/2008).

### Combination therapy study with methotrexate

Each mouse that exhibited erythema and/or swelling of one or more paws was randomly assigned to a treatment or control group and therapy was started. Mice were given a subcutaneous or intravenous injection of F8-IL10 (3 × 200 μg), saline or an intraperitoneal injection of methotrexate (3 × 100 μg). For the combination study mice were given an intravenous injection of F8-IL10 (3 × 200 μg) followed by an intraperitoneal injection of methotrexate (3 × 100 μg). Eight mice were analyzed per group. The arthritic score was evaluated daily in a nonblinded fashion. The results are displayed as the mean ± standard error for each group. Experiments were performed in agreement with Swiss regulations and under a project license granted by the Veterinäramt des Kantons Zürich, Switzerland (171/2007).

### Comparison of targeted and untargeted delivery of IL10

Cloning, expression and purification of an HyHel10-IL10 fusion protein has been described before [18]. Therapy was performed as described above. Briefly, arthritis mice were injected subcutaneously with saline, HyHel10-IL10 (200 μg), TNFRII-fusion (100 μg) or F8-IL10 (200 μg). Six to seven mice were analyzed per group. Experiments were performed in agreement with Swiss regulations and under a project license granted by the Veterinäramt des Kantons Zürich, Switzerland (171/2007).

### *Ex vivo *immunohistochemical detection of F8-IL10 and HyHel10-IL10 in arthritis paws

At the end of therapy, mice were killed and paws were embedded in cryombedding compound (Microm, Walldorf, Germany) and stored at -80°C. Sections (10 μm) were cut and fixed in acetone. F8-IL0 and HyHel10-IL10 were detected using a biotinylated anti-human IL10 antibody (eBiosciences, San Diego, CA, USA) followed by SAP complex (Biospa, Milan, Italy). Fast Red TRSalt (Sigma-Aldrich, St Louis, MO, USA) was used as the phosphatase substrate. Sections were counterstained with hematoxylin, mounted with glycergel mounting medium (Dako, Glostrup, Denmark) and analyzed with an Axiovert S100 TV microscope (Zeiss, Feldbach, Switzerland).

### Immunofluorescence studies of infiltrating cells

To evaluate the role of effector cell responses *in vivo *immunofluorescent staining of paw sections of therapy mice was performed using antibodies against the following antigens: rat anti-mouse F4/80 (anti-macrophage; Abcam, Cambridge, UK), rat anti mouse CD45 (BD Biosciences, San Jose, CA, USA), rabbit anti-asialo GM1 (anti-NK; Wako Pure Chemical Industries, Tokyo, Japan) and rat anti-mouse CD4 and rat anti-mouse CD8. Cryosections were thawed and fixed by immersion in cold acetone for 10 minutes. Blocking was performed by incubating the sections with 20% donkey/goat serum in PBS for one hour. Following washing with PBS twice for five minutes at room temperature, sections were incubated with the primary antibodies in 12% BSA in PBS over night at 4°C. Sections were washed three times for five minutes with PBS at room temperature and then incubated with fluorescent Alexa 488- or 594-coupled secondary antibodies (BD Biosciences, San Jose, CA, USA) and Hoechst, Frankfurt, Germany (4,6-diamidino-2-phenylindole) in 12% BSA-PBS. Finally, sections were washed three times for five minutes in PBS and mounted with glycergel and a coverglass (VWR International, Dietikon, Switzerland). Images were obtained using the individual fluorescent channels using an Axioskop 2 mot plus (Carl Zeiss, Feldbach, Switzerland).

Staining was quantified in representative 10 times microscopic images using ImageJ software [45] and expressed as a percentage of measurement area.

### Anti-bovine collagen-II antibodies

Levels of anti-bovine collagen-II antibodies at the termination of experiments were determined using standard ELISA techniques as described before [46]. Microtiter plates were coated with bovine collagen II solution (5 μg/ml) overnight at 4°C. After washing they were blocked for two hours at room temperature with 2% BSA. Samples were tested in triplicates at 1:800 dilution. Bound total IgG, IgG1 and IgG2a were detected by incubation with horseradish peroxidase conjugated goat anti-mouse IgG/IgG1 or IgG2a antibodies (Santa Cruz Biotechnology, Santa Cruz, CA, USA).

### Analysis of mouse plasma cytokine levels

Mouse plasma cytokine level analysis was performed at Cytolab (Cytolab, Muelligen, Switzerland). A multiplexed particle-based flow cytometric cytokine assay was used [47]. MAP Fluorokine cytokine kits were purchased from R&D (Oxon, UK). The procedures closely followed the manufacturer's instructions. The analysis was conducted using a conventional flow cytometer (FC500 MPL, BeckmanCoulter, Nyon, Switzerland).

### Toxicology studies in cynomolgus monkey

Preclinical toxicology studies were performed at Centre International de Toxicologie, Evreux, in accordance with good laboratory practice (GLP) guidelines (Study number 34975TSP).

During the study two groups (group 2 and 3) of three male and three female cynomolgus monkeys received test Dekavil (F8IL10) by subcutaneous injection in the dorsum at the dose-level of 180 μg/kg/administration, three times a week for eight weeks. Another group (group 1) of three males and three females received the formulation buffer for Dekavil (F8-IL10), under the same experimental conditions, and acted as a control group.

Animals in group 3 were also administered methotrexate starting on day 4, as well as folic acid 24 hours after each methotrexate administration. Both these test items were administered by oral gavage with capsules, once a week until the end of the study.

Blood samples were taken from all the animals for determination of serum levels of Dekavil (F8IL10) on day 1 and on the last day of dosing, at designated time-points.

Animals were checked daily for reaction to treatment and the following investigations were performed: body weight, food consumption, ophthalmoscopy, electrocardiography, blood pressure, hematology and clinical chemistry.

On completion of the treatment period, animals were sacrificed and submitted to a complete macroscopic examination.

### Single dose intravenous toxicity study in mice

Single dose toxicity study was performed at the Research Toxicology Center in Rome, Italy, in accordance with GLP guidelines (Study number 74250).

A single group of five male and five female mice (Hsd:ICR(CD-1)) was intravenously injected with 20 mg/kg F8-IL10 followed by a 14-day observation period. A control group of five male and five female mice (Hsd:ICR(CD-1)) was injected with the vehicle alone (saline). All animals were killed with carbon dioxide at the end of the observation period and subjected to necropsy.

### Statistical analysis

Data are expressed as the mean ± standard error of the mean. Differences in the arthritis score between different groups were compared using Mann-Whitney test.

## Results

### Immunohistochemical analysis of rheumatoid synovial tissue specimens

**Figure 1:**
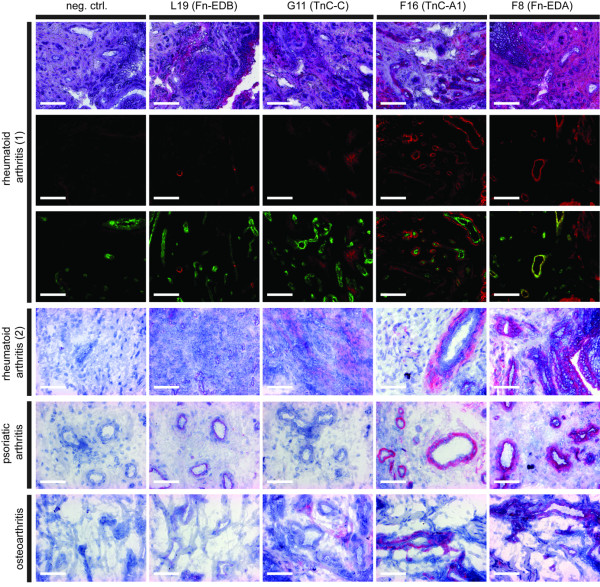
Immunohistochemical analysis of rheumatoid arthritis specimens, psoriatic arthritis specimens and osteoarthritis specimens. Immunohistochemistry with the small immunoproteins L19, G11, F16, and F8 was performed in different pathology specimens obtained from biopsies of patients with rheumatoid arthritis, psoriatic arthritis or osteoarthritis. In total, pathology specimens of seven patients were analyzed, four of them are shown above. Furthermore, immunofluorescence double staining with L19, G11, F16 and F8 (red) and von Willebrand factor (green) was performed on rheumatoid synovial tissue specimens of one patient (rheumatoid arthritis (1)). Overall F8 exhibited the strongest staining of all tested antibodies. It showed a diffuse stromal staining in certain areas and a vascular staining pattern in others. For negative controls, the primary antibody was omitted. Scale bars = 100 μm. neg ctrl = negative control.

Figure 1 presents a comparative immunohistochemical and immunofluorescence analysis of the human monoclonal antibodies L19, G11, F16 and F8. In total, pathology specimens of seven patients were analyzed, four of which are shown in Figure 1. Both F16 and F8 displayed a stronger staining pattern compared with L19 and G11. The F8 antibody sometimes exhibited a diffuse stromal staining or a vascular staining pattern, but consistently reacted strongly with both human and murine specimens of arthritis and was thus selected for pharmacodelivery applications. Furthermore, F8 and F16 exhibited a prominent perivascular staining pattern in tissue specimens from patients suffering from psoriatic arthritis and osteoarthritis. In tumor-bearing mice, the *in vivo *targeting potential of F8 and L19 was comparable when assessed by quantitative biodistribution studies [38].

### Cloning and *in vitro *characterization of F8-IL10

**Figure 2:**
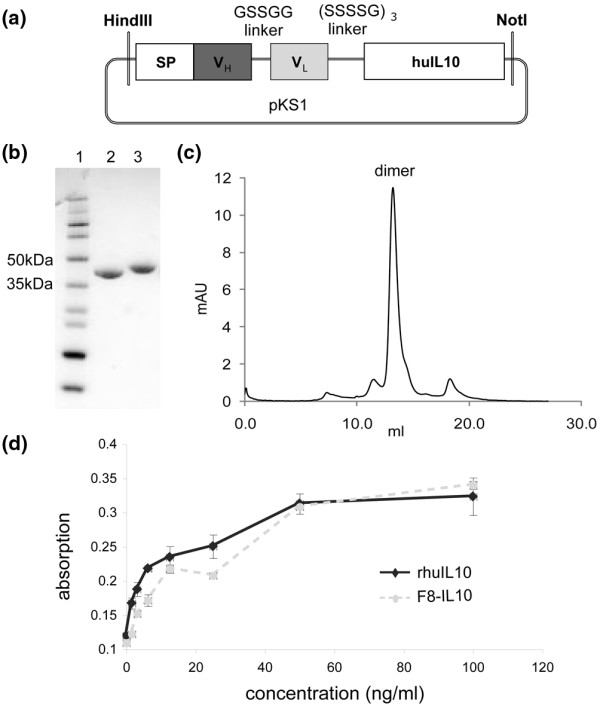
Cloning, expression and purification of F8-IL10. **(a) **Schematic representation of the cloning strategy of the F8-IL10 fusion protein. **(b) **SDS-PAGE analysis of purified fusion proteins: lane 1, molecular-weight marker; lanes 2 and 3, F8-IL10 under nonreducing and reducing conditions, respectively. **(c) **Gel-filtration analysis of affinity-purified F8-IL10. The peak eluting at a retention volume of 12 ml corresponds to the noncovalent homodimeric form of F8-IL10. **(d) **MC/9 cell proliferation assay. F8-IL10 displayed biological activity comparable with the one of recombinant human IL10 used as a standard in the assay.

The immunocytokine F8-IL10 was cloned in a mammalian expression vector by sequentially fusing the F8 in scFv format [19,38] in frame with the human IL10 gene, using flexible aminoacid linkers (Figure 2a). The resulting plasmid pKS1 was linearized and used to stably transfect CHO-S cells. A short five amino acid linker was used to bridge VH and VL domains within the scFv antibody fragment moiety, thus driving the formation of a stable non-covalent homodimer (Figures 2b and 2c) [20]. F8-IL10 could be purified to homogeneity on protein A (Figures 2b and 2c), retained full immunoreactivity when tested by affinity chromatography on an EDA-sepharose resin (data not shown) and displayed a biological activity comparable with that of recombinant human IL10 used in equimolar amounts in a MC/9 cell proliferation assay (Figure 2d) [42]. In a crossreactivity study on tissue microarray none of the healthy tissue sections showed any staining with F8-IL10, except for ovary (1/3), placenta (3/3) and uterus (2/3) [see Additional data file 1]. This finding is in excellent agreement with the known expression of oncofetal antigens in organs of the female reproductive system [48].

### F8-IL10 selectively targets arthritic lesions and tumors in mice

**Figure 3:**
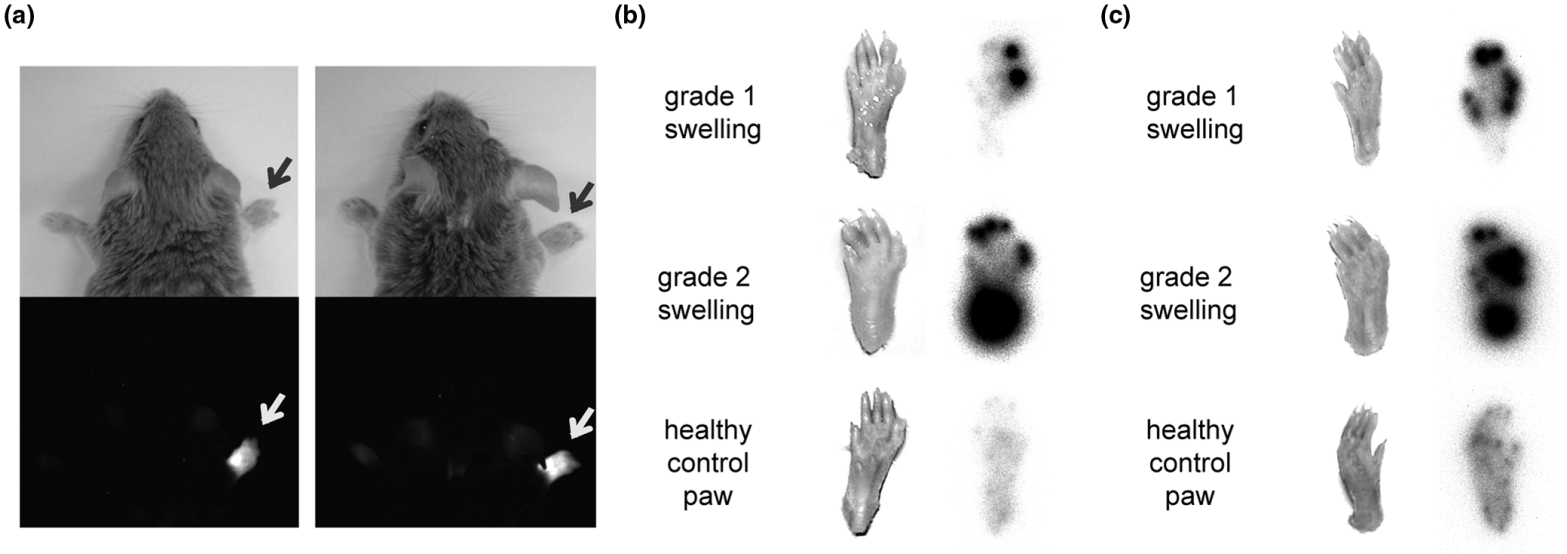
*In vivo *targeting of the small immunoprotein F8 and the fusion protein F8-IL10 in arthritic mice. **(a) **Near infrared fluorescence imaging. Arthritic mice (n = 3) were injected with small immunoprotein (SIP) (F8)-Alexa750. Near infrared fluorescence imaging analysis was performed 24 hours after injection. Arrows indicate grade 2 swelling in the front paws of the mice. **(b to c) **Phosphorimaging. Arthritic mice (n = 2) were injected (b) intravenously with ^125^I-labelled SIP(F8) or (c) subcutaneously with ^125^I-labelled F8-IL10. Uptake of radio-iodinated antibodies was analyzed by phosphorimaging 24 hours after injection. The ranges of lesion to nonaffected paw ratios measured by phosphorimaging were 7.4 to 13.9 for SIP(F8) intravenously and 5.0 to 6.8 for F8-IL10 subcutaneously.

The *in vivo *targeting properties of the F8 antibody and of F8-IL10 were tested in CIA mice, using both fluorescently labeled and radioiodinated protein preparations. Figure 3a shows near-infrared fluorescence images [44,49] of arthritic mice 24 hours after intravenous injection of 100 μg SIP(F8) [38,50] labeled with Alexa750 dye. A preferential accumulation of the F8 antibody could be detected in the inflamed extremities. A more detailed targeting analysis was obtained using ^125^I-labeled preparations of SIP(F8) and of F8-IL10. Twenty-four hours after intravenous or subcutaneous administration, arthritic limbs were imaged on a PhosphorImager, revealing a preferential protein accumulation at arthritic fingers and paws compared with healthy control paws (Figures 3b and 3c). The ranges of lesion to nonaffected paw ratios measured by phosphorimaging were 7.4 to 13.9 for SIP(F8) intravenous and 5.0 to 6.8 for F8-IL10 subcutaneous. The administration of comparable amounts of antibodies of irrelevant specificity in the mouse in recombinant SIP format did not exhibit any preferential uptake at sites of inflammation [51].

**Figure 4:**
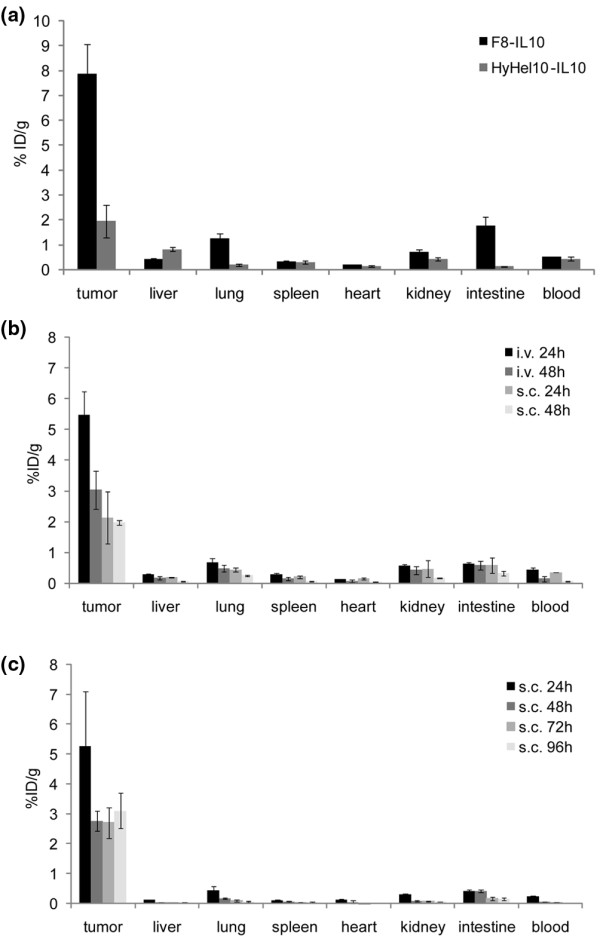
Biodistribution study in F9 tumor-bearing mice. In all biodistribution experiments four mice were analyzed per group. Radioactivity content of tumor or organs is expressed as percentage of the injected dose per gram of tissue (%ID/g) ± standard error. **(a) **Comparison of intravenous and subcutaneous injection. Tumor bearing mice were injected intravenously or subcutaneously with ^125^I-labelled F8-IL10 and sacrificed 24 or 48 hours after injection. **(b) **Comparison of targeted and untargeted IL10. Mice were injected intravenously with ^125^I-labelled F8-IL10 or ^125^I-labelled HyHel10-IL10 (HyHel10 is an antibody specific to hen egg lysozyme and is not recognizing any murine antigen). They were sacrificed 24 hours after injection. **(c) **Residence time of F8-IL10 following subcutaneous administration. Mice were injected with ^125^I-labelled F8-IL10 and sacrificed 24, 48, 72, or 96 hours after injection.

The subcutaneous administration of therapeutic proteins in patients with arthritis is often preferable compared with the intravenous administration route, which is typically performed at the hospital. In order to investigate whether a selective *in vivo *targeting of lesions could be obtained using F8-IL10 both with subcutaneous and intravenous administrations, we performed a comparative biodistribution study in tumor-bearing mice. We chose a cancer model rather than an arthritis model for this analysis, because tumor-bearing mice provide a quantitative biodistribution analysis of therapeutic proteins. Figure 4a illustrates biodistribution results (expressed as a percentage of injected dose per gram of tissue) for a radioiodinated preparation of F8-IL10, administered intravenously or subcutaneously. For both administration routes, a preferential tumor uptake could be observed, with excellent tumor:organ ratios at 24 and 48 hours following injection. An antibody-IL10 fusion protein of irrelevant specificity in the mouse [1,50,52] exhibited a reduced tumor uptake in the same animal model (Figure 4b). In order to quantitatively assess the residence time of F8-IL10 on neovascular lesions following subcutaneous administration, a biodistribution study was performed sacrificing tumor-bearing mice at 24, 48, 72 and 96 hours and correcting for the tumor volume increase during the study period. Figure 4c shows that the immunocytokine efficiently and stably localized at the tumor site, while being cleared from all normal organs. No statistically significant difference could be observed in terms of tumor uptake between the 48 and 96 hour time points.

### Inhibition of arthritis progression in the collagen-induced model of arthritis

The CIA model was used to assess the therapeutic potential of F8-IL10 when used alone or in combination with methotrexate. Mice were allowed to reach an arthritic score of 1 to 3, before receiving three injections (days 1, 4 and 7) of F8-IL10 (200 μg) and/or of methotrexate (100 μg). The F8-IL10 dose for the mouse was calculated from the recommended equivalent dose of 20 μg/kg of recombinant human IL10 used in clinical trials using a body surface correction algorithm [53] and a correction factor for the activity of human IL10 in mice [29].

**Figure 5:**
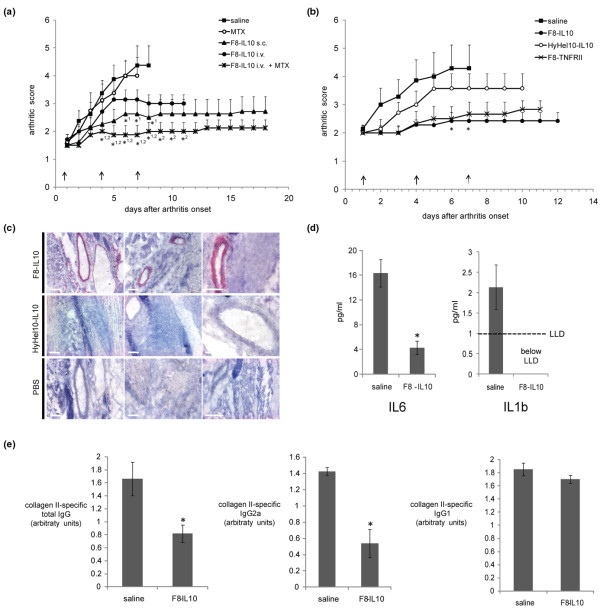
Therapy studies of F8-IL10 in the CIA mouse model. **(a) **Combination with methotrexate. Arthritic mice were given injections with saline (black squares), methotrexate 100 μg intraperitoneally (open circles), F8-IL10 200 μg subcutaneously (black triangles), F8-IL10 200 μg intravenously (black circles), or a combination of F8-IL10 200 μg intravenously and methotrexate (MTX) 100 μg intraperitoneally (crosses). Injections were started at day 1 after arthritis onset and then repeated every third day for three injections per animal, as indicated by the arrows. The arthritic score was evaluated daily and expressed as the mean ± standard error of the mean (SEM) of eight mice per group. * ^1 ^*P *< 0.05 versus saline; * ^2 ^*P *< 0.05 versus F8-IL10 intravenously. **(b) **Comparison of targeted versus systemic application of IL10. Arthritic mice were injected subcutanously with saline (black squares), HyHel10-IL10 200 μg (open circles), F8-TNFRII (crosses), or F8-IL10 200 μg (black circles) every third day for three injections, as indicated by arrows. Arthritic score is expressed as the mean ± SEM of six to seven mice per group. * *P *< 0.05 versus saline. **(c) ***Ex vivo *immunohistochemical detection of F8-IL10 and HyHel10-IL10 in arthritis paws. Analysis of the arthritis paws at the end of therapy (day 12 for F8-IL10 and day 10 for HyHel10-IL10) showed that F8-IL10 is still detectable by immunohistochemistry using an anti-human-IL10-antibody. **(d) **Analysis of plasma cytokines levels at the end of therapy. F8-IL10-treated mice showed significantly decreased IL6 levels compared with the saline group. Furthermore, IL1b serum levels of F8-IL10-treated mice were below the lower limit of detection. * *P *< 0.05 versus saline. **(e) **Anti type-II collagen antibodies. Titers of bovine type II collagen-specific total IgG, IgG1 and IgG2a antibodies were determined by ELISA. A clear reduction of total IgG and IgG2a, but not IgG1, antibody levels was observed in F8-IL10-treated mice. * *P *< 0.05 versus saline.

Figure 5a shows that mice treated with methotrexate did not exhibit any detectable reduction of arthritis, in line with previously published results where comparable doses of methotrexate in the same mouse model had no significant effect on the onset of CIA [54]. Disease progression was substantially inhibited for F8-IL10 with intravenous administration and with subcutaneous administration. Both subcutaneous injections of F8-IL10 and the combination treatment of methotrexate plus intravenous F8-IL10 allowed the maintainence of an arthritic score below 3 until the mice were sacrificed (18 days after the beginning of pharmacological treatment). Similar to what has previously been reported [18], the therapeutic performance of an antibody-IL10 fusion protein of irrelevant specificity in the mouse exhibited a worse therapeutic benefit, confirming the contribution of selective targeting to therapeutic outcome (Figure 5b). We were not allowed by the local authorities (Veterinäramt des Kantons Zürich) to extend the duration of the observation period for the mice in order to keep animal discomfort within an acceptable limit, but it would have obviously been of scientific interest to monitor disease stabilization over a longer period of time.

Paws and blood of mice were analyzed at the end of the therapy and we could demonstrate by immunohistochemistry that F8-IL10 is still detectable in arthritic paws (Figure 5c). Analysis of plasma cytokines of sacrificed mice showed significantly (*P *= 0.004) decreased IL6 levels for F8-IL10-treated mice (Figure 5d). Furthermore, saline-treated mice showed elevated IL1b levels compared with healthy control and F8-IL10-treated mice. In our mouse model of CIA, the therapeutic activity of F8-IL10 was found to be comparable with the one of a recombinant biopharmaceutical based on the extracellular part of murine TNF receptor 2, administered with the same schedule (Figure 5b).

**Figure 6:**
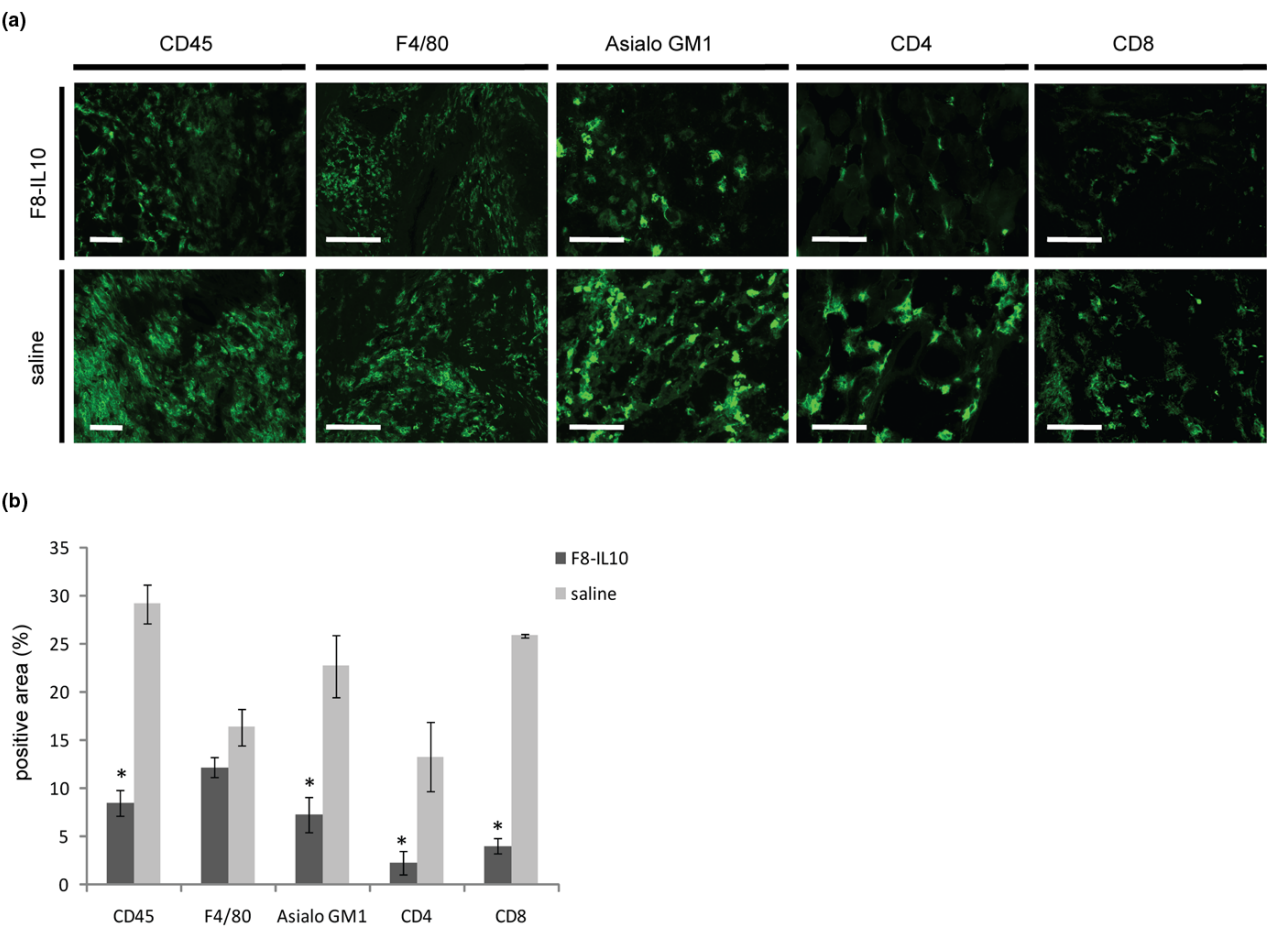
Immunofluorescence analysis of infiltrating cells. At termination of the therapy experiment a comparative immunofluorescence analysis of infiltrating cells from mice treated with saline or F8-IL10 was performed. **(a) **Representative immunofluorescence images of paw sections. Scale bars = 100 μm. **(b) **Sections were evaluated for area percentage positive staining and a significant decrease of infiltrating leukocytes was observed. * *P *< 0.05 versus saline.

Figure 6 shows a comparative immunofluorescence analysis of infiltrating cells from mice treated with saline or F8-IL10. Staining with an anti-CD45 antibody revealed that F8-IL10-treated mice presented a significantly (*P *= 0.03) lower level of infiltrating leukocytes in the paw compared with the saline treatment group. In accordance with this finding, staining with an anti-asialo-GM1 antibody, which preferentially stains natural killer cells, with the macrophage-specific antibody F4/80 and with CD4/CD8 antibodies, showed a decreased infiltration of these cells in paws of F8-IL10-treated mice.

### Anti type-II collagen antibodies

Humoral immunity was followed by measurement of serum levels of anti-collagen II immunoglobulin (Ig) isotypes. Serum samples were obtained from both control and F8-IL10-treated animals at the termination of the experiment and total IgG antibody levels, as well as IgG1 and IgG2a isotype levels were determined by ELISA (Figure 5e). Total IgG levels were significantly lower in F8-IL10-treated animals than in controls (*P *< 0.05). When analyzing specific isotypes, no significant differences were seen in the anti-collagen II IgG1 levels between the two groups. However, the anti-collagen II IgG2a titers were significantly lower (*P *< 0.05) in sera from F8-IL10-treated mice, as seen for other anti-arthritic therapeutic interventions in the CIA model [55].

### Safety pharmacology profile of F8-IL10

In preparation for a dose-finding, pharmacokinetic phase I study of F8-IL10 in combination with methotrexate in patients with active rheumatoid arthritis we performed a toxicity assessment of F8-IL10 in combination with methotrexate in cynomolgus monkeys. In this study, three groups of monkeys (each group consisting of three female and three male animals) received administrations of either F8-IL10 alone, F8-IL10 plus methotrexate or saline. During the study F8-IL10 was injected subcutaneously three times a week for eight weeks at a dosage of 180 μg/kg (60 μg/kg IL10 equivalents), which reflects 10 times the initial human dose intended for administration during the phase I clinical study. Methotrexate was given on a weekly basis at the standard dosage of 0.65 mg/kg.

There were no relevant findings in body weight evolution, food consumption, quantitative electrocardiography parameters or systolic and diastolic blood pressure values. No relevant ophthalmological findings were noted in any groups. A ventricular premature complex was recorded in one female treated with F8-IL10 alone in week 4, after treatment.

During the course of the study (week 4), a regenerative anemia was observed, however a complete recovery was noted in week 7. No toxicologically relevant findings were observed in the blood biochemical parameters at the end of week 4 and at the end of the treatment period in any groups.

Pharmacokinetic data were obtained during the toxicology study. Blood samples were collected at pre-dose, 5 and 30 minutes, and 3 and 24 hours after the injection. The serum concentration of F8-IL10 was measured using a validated colorimetric ELISA. Many of the samples analyzed were found to be below the level of quantification (< 0.25 ng/ml). However, for those samples in which a positive result was obtained, maximum serum levels were generally observed three hours after the subcutaneous injection of F8-IL10 with serum levels of about 20 ng/ml. After 24 hours no more detection of F8-IL10 in serum was possible.

In conclusion, subcutaneous administration of F8-IL10 alone or in combination with methotrexate was generally well tolerated.

The acute toxicity of F8-IL10 was investigated in mice after intravenous administration of a single dose level of 20 mg/kg, corresponding to 300 times the human starting dose proposed for clinical trials [53], followed by a 14-day observation period. Body weights were recorded weekly and necropsy was performed on all animals. No mortality occurred and no clinical signs were noted in both male and female animals. Changes in body weight observed at the end of the study were within the expected range for this strain and age of animals. No changes of toxicological significance were observed in the weight of organs. No abnormalities were detected in all treated animals at the necropsy examination and no abnormalities were observed at the injection site.

These results indicate that F8-IL10 had no toxic effect on mice following a single intravenous administration at a dose level of 20 mg/kg body weight. The product was well locally tolerated when injected into the tail vein at the dose level tested.

## Discussion

In this article, we have compared four human monoclonal antibodies specific to alternatively-spliced components of the extracellular matrix and have identified F8 as a suitable candidate for pharmacodelivery applications in rheumatoid arthritis. F8 recognizes the extra-domain EDA of fibronectin [38] and consistently yielded stronger staining of arthritic specimens compared with the L19, G11 and F16 antibodies. In analogy to our previous work in this area [18], we fused F8 to human IL10, generating the immunocytokine F8-IL10 (DEKAVIL), which was shown to preferentially localize at sites of arthritis in the collagen-induced murine model of the disease. F8-IL10 was able to stabilize clinical features of arthritis in this animal model and was found to be well tolerated in monkeys at human equivalent doses of 20 μg/kg [53].

Preclinical studies were facilitated by the fact that F8 binds with comparable affinity to EDA of murine, monkey and human origin [38].

The rationale behind the development of F8-IL10 as a novel biopharmaceutical relies on the promising, yet not sufficiently satisfactory, preclinical and clinical data reported for recombinant human IL10 (Tenovil TM). In controlled clinical trials in patients with rheumatoid arthritis, Tenovil exhibited ACR20 values substantially higher than the ones in control groups and comparable with the ACR20 values reported for TNF blockers. However, the ACR50 values observed with Tenovil, while significantly better compared with the ones observed in patients treated only with methotrexate, were not as good as those reported for Humira (Adalimumab), Remicade (Infliximab) and Enbrel (Etanercept) [32-34].

In spite of these observations, we and others have extensively demonstrated in animal models that the antibody-based delivery of cytokines to sites of disease can substantially improve the therapeutic index of these biopharmaceuticals. Indeed, our group has developed fully human fusion proteins based on the pro-inflammatory cytokines IL2 and TNF (L19-IL2; L19-TNF; F16-IL2) [3,8,10,11] which are currently being investigated in phase I and in phase II clinical trials in patients with cancer. To our knowledge, F8-IL10 will be the first anti-inflammatory immunocytokine to be tested in the clinical setting and it will be interesting to learn whether the improved performance and selectivity documented in the mouse model of arthritis holds true for patients with rheumatoid disease. Encouraged by the excellent tolerability profile observed in cynomolgus monkeys, we have submitted a request for clinical trials in Italy.

When developing F8-IL10 for industrial pharmaceutical programs, care was devoted to identifying a suitable formulation which could be compatible with subcutaneous administration. Indeed, we were not aware at the beginning of the study of any quantitative biodistribution analysis performed with disease-targeting antibody fragments following subcutaneous administration. Using radioiodinated protein preparations, we studied the biodistribution properties of F8-IL10 both in mouse models of arthritis and in tumor-bearing mice, where targeting performance can be expressed as percent injected dose per gram. The conventional intravenous administration route yielded tumor targeting results comparable with the ones obtained following a subcutaneous administration, thus providing a robust rationale for the development of clinical trials featuring subcutaneous injections. Experience gained with TNF blocking antibodies suggests that subcutaneous administration may be better accepted by patients and may lead to a better compliance, reducing the need to visit hospital sites for each administration.

## Conclusions

The data presented in this article provide a strong rationale for the clinical investigation of F8-IL10 as a novel biopharmaceutical for the therapy of patients with rheumatoid arthritis who have failed at least two lines of biological therapy. Clinical studies will reveal whether the promising preclinical results can be translated to the clinical setting and, potentially, whether F8-IL10 could find a broader clinical applicability as a targeted anti-inflammatory agent for diseases which over-express the EDA domain of fibronectin.

## Abbreviations

ACR: American College of Rheumatology; BSA: bovine serum albumin; CIA: collagen-induced arthritis; DMSO: dimethylsulfoxide; EDA: extra-domain A of fibronectin; EDB: extra-domain B of fibronectin; ELISA: enzyme linked immunosorbent assay; GLP: good laboratory practice; HyHel: antibody specific to hen egg lysozyme; Ig: immunoglobulin; IL: interleukin; PBS: phosphate buffered saline; PCR: polymerase chain reaction; rhuIL10: recombinant human IL10; SAP: streptavidin-alkaline phosphatase; scFv: single chain variable fragment; SIP: small immunoprotein; TnC: tenascin C; TNF: tumor necrosis factor.

## Competing interests

DN is a cofounder and shareholder of Philogen SpA (Siena, Italy), the company that owns DEKAVIL.

## Authors' contributions

KS participated in designing the study, cloned, produced and characterized the F8-IL10 fusion protein, performed the animal experiments and assisted in preparing the manuscript. MK and ET participated in characterizing the fusion proteins and assisted in the animal experiments. FB set up the animal model in our laboratory and contributed essentially to the animal experiments. RM and EP provided the human arthritic specimens and gave helpful advice. DN and ET supervised the experiments, were involved in data interpretation and prepared the manuscript. All authors read and approved the final manuscript.

## Supplementary Material

Additional file 1 A Figure showing crossreactivity of F8-IL10 study on tissue microarray sections (Biochain, Hayward, USA). Sections were blocked with FCS and then incubated with 5 μg/ml of purified FITC-labeled F8-IL10 for one hour. For amplification of the signal bound antibody was detected using rabbit anti-FITC antibody and subsequent AlexaFluor594 goat anti-rabbit IgG. Slides were mounted with glycergel and analyzed with an AxioScop 2MOT+ fluorescence microscope. None of the healthy tissue sections showed any staining with F8-IL10, except for ovary (1/3), placenta (3/3) and uterus (2/3). Click here for file
