# Supplementary figures and images for: Preclinical characterization of DEKAVIL (F8-IL10), a novel clinical-stage immunocytokine which inhibits the progression of collagen-induced arthritis
Source: Arthritis Res Ther. 2009 Sep 25;11(5):R142. doi: 10.1186/ar2814 (PMC2787264; doi:10.1186/ar2814)

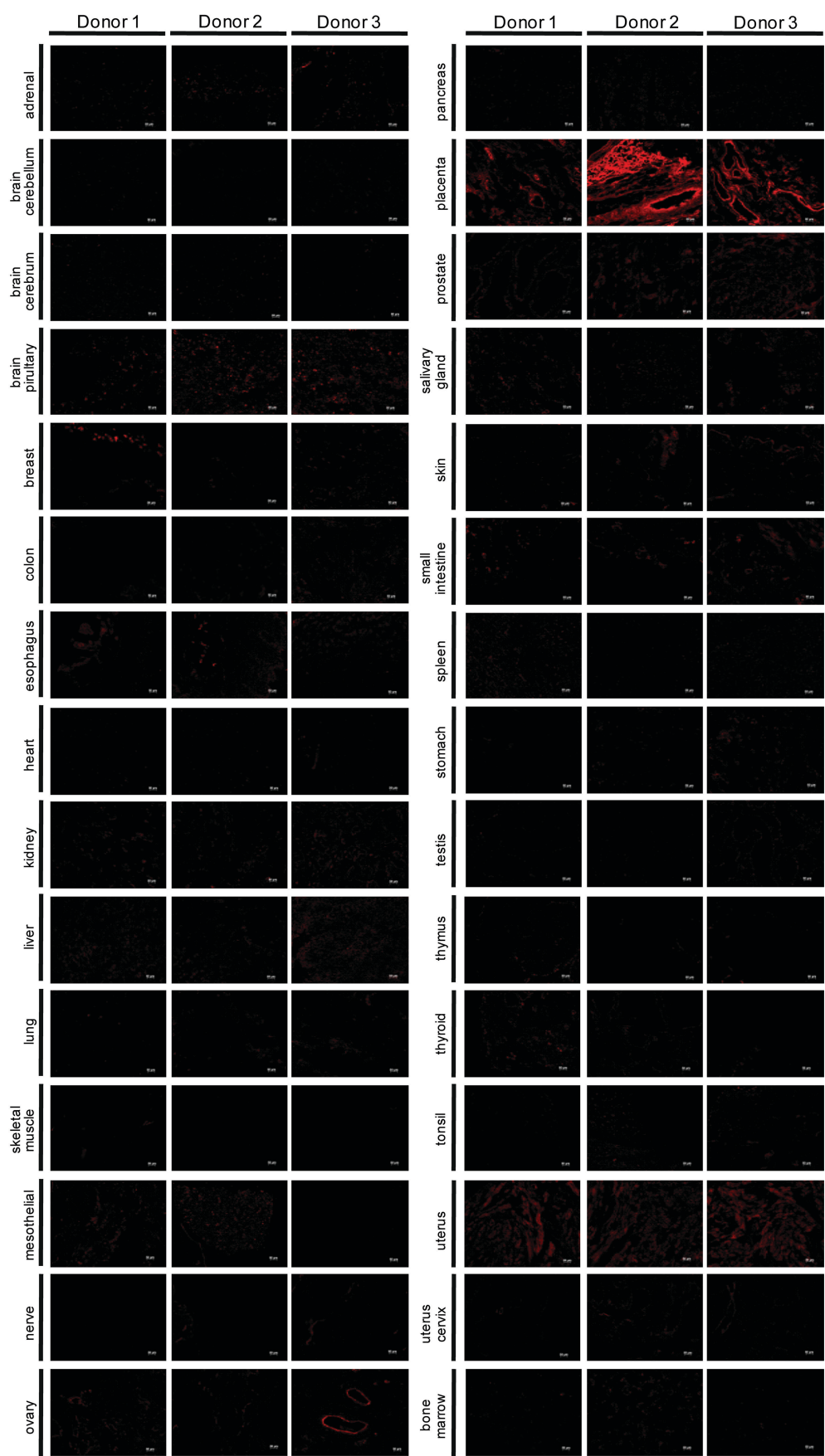

Supplement: Additional file 1 — A Figure showing crossreactivity of F8-IL10 study on tissue microarray sections (Biochain, Hayward, USA). Sections were blocked with FCS and then incubated with 5 μg/ml of purified FITC-labeled F8-IL10 for one hour. For amplification of the signal bound antibody was detected using rabbit anti-FITC antibody and subsequent AlexaFluor594 goat anti-rabbit IgG. Slides were mounted with glycergel and analyzed with an AxioScop 2MOT+ fluorescence microscope. None of the healthy tissue sections showed any staining with F8-IL10, except for ovary (1/3), placenta (3/3) and uterus (2/3). [file ar2814-S1.PDF]
